# 2,4-Diaminopyrimidines as Potent Inhibitors of *Trypanosoma brucei* and Identification of Molecular Targets by a Chemical Proteomics Approach

**DOI:** 10.1371/journal.pntd.0000956

**Published:** 2011-02-08

**Authors:** Luke Mercer, Tana Bowling, Joe Perales, Jennifer Freeman, Tien Nguyen, Cyrus Bacchi, Nigel Yarlett, Robert Don, Robert Jacobs, Bakela Nare

**Affiliations:** 1 SCYNEXIS Inc., Research Triangle Park, North Carolina, United States of America; 2 Haskins Laboratories, Pace University, New York, New York, United States of America; 3 Drugs for Neglected Diseases initiative (DNDi), Geneva, Switzerland; Institute of Tropical Medicine, Belgium

## Abstract

**Background:**

There is an urgent need to develop new, safe and effective treatments for human African trypanosomiasis (HAT) because current drugs have extremely poor safety profiles and are difficult to administer. Here we report the discovery of 2,4-diaminopyrimidines, exemplified by 4-[4-amino-5-(2-methoxy-benzoyl)-pyrimidin-2-ylamino]-piperidine-1-carboxylic acid phenylamide (SCYX-5070), as potent inhibitors of *Trypanosoma brucei* and the related trypanosomatid protozoans *Leishmania spp*.

**Methodology/Principal Findings:**

In this work we show that loss of *T. brucei* viability following SCYX-5070 exposure was dependent on compound concentration and incubation time. Pulse incubation of *T. brucei* with SCYX-5070 demonstrates that a short period of exposure (10–12 hrs) is required to produce irreversible effects on survival or commit the parasites to death. SCYX-5070 cured an acute trypanosomiasis infection in mice without exhibiting signs of compound related acute or chronic toxicity. To identify the molecular target(s) responsible for the mechanism of action of 2,4-diaminopyrimidines against trypanosomatid protozoa, a representative analogue was immobilized on a solid matrix (sepharose) and used to isolate target proteins from parasite extracts. Mitogen-activated protein kinases (MAPKs) and cdc2-related kinases (CRKs) were identified as the major proteins specifically bound to the immobilized compound, suggesting their participation in the pharmacological effects of 2,4-diaminopyrimidines against trypanosomatid protozoan parasites.

**Conclusions/Significance:**

Results show that 2,4-diaminopyrimidines have a good *in vitro* and *in vivo* pharmacological profile against trypanosomatid protozoans and that MAPKs and CRKs are potential molecular targets of these compounds. The 2,4-diminipyrimidines may serve as suitable leads for the development of novel treatments for HAT.

## Author Summary

The protozoan parasite *Trypanosoma brucei* is the causative agent of human African trypanosomiasis (HAT) or sleeping sickness, a fatal disease affecting nearly half a million people in sub-Saharan Africa. Current treatments for HAT have very poor safety profiles and are difficult to administer. There is an urgent need for new, safe and effective treatments for sleeping sickness. This work describes the discovery of 2,4-diaminopyrimidines, exemplified by 4-[4-amino-5-(2-methoxy-benzoyl)-pyrimidin-2-ylamino]-piperidine-1-carboxylic acid phenylamide or SCYX-5070, as potent inhibitors of *T. brucei* growth *in vitro* and also in animal models for HAT. To determine the parasite proteins responsible for interaction with SCYX-5070 and related compounds, affinity pull-downs were performed followed by sequence analysis and parasite genome database searching. The work revealed that mitogen-activated protein kinases (MAPKs) and cdc2-related kinases (CRKs) are the major proteins specifically bound to the immobilized compound, suggesting their potential participation in the pharmacological effects of 2,4-diaminopyrimidines against trypanosomatid protozoan parasites. These data strongly support the use of 2,4-diminipyrimidines as leads for the development of new drug candidates for the treatment of HAT.

## Introduction

Human African trypanosomiasis (HAT) or sleeping sickness is caused by the trypanosomatid protozoan parasite *Trypanosoma brucei*. The two main trypanosome species that cause HAT are *T. b. gambiense* and *T. b. rhodesiense*. An estimated 50 million people in more than 20 sub-Saharan African countries are at risk of HAT. Although the number of new cases of HAT have been declining steadily over the years, approximately 10,000 cases were reported in 2009 [1]. Trypanosome parasites are transmitted to humans through a bite from the tsetse fly. When left untreated, trypanosomes invade the central nervous system (CNS) causing a second stage of the disease (stage 2) which leads to coma and death.

Current treatment options for HAT are very limited and not ideal due to toxicity or lengthy parenteral administration. During the first stage (stage 1) of the disease when parasites are limited to the hemolymphatic system, treatment with the non-CNS penetrating drugs pentamidine (*T. b. gambiense*) or suramin (*T. b. rhodesiense*) is effective. For the CNS form of the disease (stage 2), patients are treated with the arsenic derivative melarsoprol or eflornithine, which is only effective against the subspecies *T. b. gambiense* [2]. Melarsoprol is particularly toxic because it causes reactive encephalopathy in 5–10% of the patients, and 50% of these patients die from this adverse reaction [3], [4]. Incidence of melarsoprol treatment failures of up to 25% have been reported in endemic areas [3], [5]. Prospects for vaccine development are very limited [6]; therefore, chemotherapy remains the major component of HAT control efforts. A safe oral drug that is effective against both stage 1 and stage 2 HAT would eliminate the need for disease staging and raise prospects of elimination of sleeping sickness.

We have initiated a drug discovery project to identify and develop new and effective treatments for HAT. In this report, we describe discovery and characterization of the pharmacological properties of 2,4-diaminopyrimidines against *T. brucei*. Original 2,4-diaminopyrimidine hits were identified from a high throughput screening of SCYNEXIS proprietary libraries. This was followed by hit-to-lead and lead optimization activities that resulted in the discovery of the optimized lead SCYX-5070 described in this work. Related diaminopyrimidines have previously been described as potent and selective ATP competitive inhibitors of human cyclin-dependent kinases (CDKs) for potential treatment of cancer [7], [8]. Here, we show that 2,4-diaminopyrimidines have a rapid time and concentration dependent cidal activity against *T. brucei in vitro* and cure mice with the acute form (stage 1) of trypanosomiasis. Activity was also demonstrated *in vitro* against the related trypanosomatid protozoan parasites *Leishmania donovani* and *L. major*. Because the activity of 2,4-diaminopyrimidines versus *T. brucei* was discovered and optimized using phenotypic screens, we have conducted studies to identify potential molecular targets that may be responsible for the mechanism of action against these parasites. Immobilized affinity approaches have previously been used to identify targets of antiparastic compounds [9]–[11]. Using a similar chemical proteomics approach involving immobilized 2,4-diaminopyrimidine affinity chromatography and mass spectrometry, we identified mitogen-activated protein kinases (MAPKs) and cdc2-related kinases (CRKs) as potential targets. Taken together, these findings suggest that kinetoplastid kinases offer an attractive opportunity to develop safe and effective therapy against HAT and leishmaniasis.

## Materials and Methods

### Ethics Statement

All animals used in this work were conventionally housed in facilities accredited by the Association for Assessment and Accreditation of Laboratory Animal Care and were provided food and water ad libitum. Animal studies were conducted in accordance with the recommendations in the Guide for the Care and Use of Laboratory Animals of the National Institutes of Health. Protocols for efficacy studies were approved by the Institutional Animal Care and Use Committee of Pace University (Animal Assurance Welfare Number: A3112-01). Surviving animals were euthanized by carbon dioxide asphyxiation in sealed containers as approved by the American Veterinary Association. Protocols for infection and recovery in rats were approved by the Institutional Animal Care and Use Committee of University of North Carolina, Chapel Hill.

### Parasites and cell culture

The bloodstream-form *T. brucei brucei* 427 strain was obtained from Dr. Ken Stuart (Seattle Biomedical Research Institute, Seattle, WA). Parasites were cultured in complete HMI-9 medium [12] containing 10% FBS, 10% serum plus (SAFC Biosciences, KS), 100 units penicillin and 0.1 mg/mL streptomycin and kept in humidified incubators at 37°C and 5% CO_2_. Procyclic *T. b. brucei* strain 29-13 was obtained from Dr. James Morris (Clemson University, Clemson, SC) and cultured at 27°C with 5% CO_2_ using semi-defined medium (SDM-79) supplemented with 10% FBS [13]. *In vivo* derived bloodstream-form *T. b. brucei* 427 parasites were isolated from adult female Wistar rats that were infected by intraperitoneal injection with 1×10^5^ *T. brucei* obtained from tissue culture. Rat blood was collected at peak parasitemia (4–5 days post-infection) by cardiac puncture under terminal anesthesia and parasites were recovered using DEAE cellulose (Sigma-Aldrich, St. Louis, MO) anion-exchange columns as previously described [14].

Promastigote stages of *L. major* (FV1 strain/MHOM/IL/80/Friedlin) and *L. donovani* (LdBob strain/MHOM/SD/62/1SCL2D) were provided by Dr. Stephen Beverley (Washington University, St. Louis, MO). *Leishmania* promastigotes were cultured at 26°C in M199 medium supplemented with 10% FCS [15], [16]. Axenic amastigotes of *L. donovani* were generated by transformation from promastigotes at 37°C and low pH as previously described [15], [17]. To ensure log growth phase, protozoan parasites were sub-cultured at appropriate dilutions (typically 1: 0–100) in fresh medium every 3–4 days. L929 mouse fibroblast cells (ATCC CCL1) from American Type Culture Collection (Rockville, MD) were used to determine parasite versus mammalian cell selectivity. Fibroblasts were cultured in DMEM, supplemented with 10% fetal bovine serum, 100 units penicillin and 0.1 mg/mL streptomycin.

### *In vitro* compound sensitivity assays

To prepare for assay, *T. brucei* and *Leishmania spp.* parasites in log phase growth were counted and diluted to 2×10^5^/mL in their specified media to generate a 2-fold working concentration for the assay. Compounds to be tested were serially diluted in dimethyl sulphoxide (DMSO) and 0.5 µL added to 50 µL media (triplicate) in 96-well plates using a Biomek NX liquid handler (Beckman Coulter, Fullerton, CA). Parasites from the diluted stock were added to each well (50 µL) using a Multidrop 384 dispenser (Thermo Electron Corporation) to give a final concentration of 1×10^5^/mL parasites, and the final DMSO concentration was ∼0.5%. For evaluation of mammalian cell cytotoxicity, L929 fibroblasts were seeded at 2,000 cells/well and exposed to 2-fold dilutions of test compounds directly parallel to the assay for parasitic activity. Plates with parasites or L929 cells were incubated with compounds under appropriate conditions for each cell type. After 72 h of incubation, resazurin (Sigma-Aldrich, St. Louis, MO; 20 µL of 12.5 mg/mL stock in phosphate buffered saline) was added to each well and plates were incubated for an additional 4–6 h. To assess cell viability, fluoroscence was read in the EnVision plate reader (Perkin Elmer, Waltham, MA) at an excitation wavelength of 530 nm and emission of 590 nm. Triplicate data points were averaged and XLfit software (fit model 205) from IDBS (Guilford, UK) was used to generate sigmoidal dose-response curves for the determination of IC_50_ or IC_90_ values. The minimum inhibitory concentration (MIC), defined as the lowest concentration of compound that completely inhibits visible parasite growth, was determined by visual inspection of 96-well plates after 48–72 h of incubation with test compounds.

### Time kill assays

Determination of compound-mediated killing of *T. brucei in vitro* over time (i.e. time kill) was conducted using the CellTiter-Glo reagent (Promega Inc., Madison, WI) to measure parasite ATP content as a real time indicator of viability. Test compounds were serially diluted into white-wall/clear-bottom 96-well plates (Corning Inc. Life Sciences, Lowell, MA) containing HMI-9 media (50 µL/well) and parasites were added at a concentration of 2×10^5^/mL (50 µL/well) to duplicate plates for each evaluation time point. At the selected time, 45 µL of CellTiter-Glo reagent was added to a set of duplicate plates. The contents of the wells were mixed to lyse the parasites and plates were incubated in the dark for 10 min and read on the EnVision plate reader using a luminescence protocol to measure cellular ATP content.

### Tests for reversibility of trypanocidal effects

To establish the time required to cause persistent or irreversible effects by test compounds, *T. brucei* parasites were assessed for their ability to recover from transient exposure to test compounds. Parasites were seeded in 96-well plates at 1×10^6^/mL (100 µL/well) and incubated with serially diluted test compound. At the desired time (exposure time) parasites were spun at 4,400 rpm for 5 min and the supernatant was aspirated to remove the compound. The wash process was repeated two more times to remove residual compound and parasites were incubated in drug-free HMI-9 media. Following a 72 h incubation in drug free medium, resazurin was added and parasite viability was determined as described for the *in vitro* sensitivity assay.

### Efficacy in mouse model for acute HAT

For efficacy studies against acute infections, groups of 3 female Swiss Webster mice (Taconic Farms, Germantown, NY) were injected intraperitoneally (i.p.) with infected rat blood containing 500 trypanosomes (*T. b. brucei* EATRO 110 strain). The infection was allowed to progress for 24 hrs prior to treatment with test compound given daily by bolus i.p. injection or oral gavage for 4 consecutive days. Test compounds were formulated in 20% ethanol, 50% polyethylene glycol 400 and 30% carboxymethyl cellulose and given at 200 µL for a 25 g animal. Animals were monitored daily for signs of compound toxicity and clinical signs of trypanosomiasis for a period of 30 days. After the end of the dosing period, mice were checked for parasitemia twice a week by microscopic examination of smears prepared from tail vein blood. Animals remaining parasite free for more than 30 days beyond the end of the treatment period were considered cured [18]. Control untreated animals typically succumbed to the infection within 7–8 days following i.p. inoculation with parasites.

### Compound synthesis and immobilization

All test compounds were synthesized and supplied by the medicinal chemistry team at SCYNEXIS Inc. For compound immobilization, epoxy activated Sepharose 6β (GE Healthcare, Piscataway, NJ) (4.0 g, 19–40 µ mol/gram) was washed with distilled water (0.80 L); then moist beads were transferred to a scintillation vial and suspended in DMF/water 1∶1 (8.0 mL). A solution of 4-[4-amino-5-(2-methoxy-benzoyl)-pyrimidin-2-ylamino]-piperidine-1-carboxylic acid (4-hydroxy-phenyl)-amide or SCYX-7434 (11 mg, 6 µmol/gram beads) in DMF/water 1∶1 (4.0 mL) was added to the suspension and pH adjusted to 11 with aqueous NaOH [0.1]. The reaction was shaken at 40°C for 16 h before a solution of ethanolamine (0.14 mL, 2.4 mmol) in DMF/water 1∶1 (2.4 mL) was added and allowed to shake at 40°C for an additional 24 h. Compound-immobilized bead suspension was filtered on a Buchner funnel and washed with DMF/water 1∶1, [0.1] aq. NaHCO_3_, [0.5] aq NaCl, [0.1] aq. NaCl (adjusted to pH 8.0), [0.1] aq NaOAc and pH 4.0 aqueous buffer. The remaining solid was suspended in pH 7.2 PBS buffer (2X volume of solid). Control beads were generated by blocking reactive groups by reaction with ethanolamine in DMF/water 1∶1 at 40°C with shaking for 24 h. Control beads were then filtered, washed and resuspended as described above. Compound-immobilized or control blocked sepharose beads were stored at 4°Cas a 50% slurry in phosphate buffer pH 7.4 containing 0.2% sodium azide.

### Cell lysis, affinity chromatography and gel electrophoresis

The protozoan parasites were cultured under conditions described earlier, washed several times with phosphate buffered saline, and the final pellets were stored at −80°C until protein extraction and affinity association were performed. Procedures for extract preparation and affinity chromatography were based on work done previously by others [19], [20] with some modifications. Frozen parasite pellets were suspended in lysis buffer (LB) containing 20 mM HEPES, pH 7.5, 50 mM NaCl, 0.25% NP-40, 1 mM EDTA, 1 mM EGTA, 1 mM DTT, 10% glycerol and Complete protease inhibitor cocktail (Roche Applied Science, Indianapolis, IN). Protein concentrations were estimated based on correlations with cell counts and protein measurements using the Bio-Rad assay (Bio-Rad, Hercules, CA). Parasite pellets containing 0.5 mL/5 mg protein were sonicated while on ice with 2×5 sec pulses on a Branson Sonifier 150 (Branson, Danbury, CT). Lysates were centrifuged at 10,000×g at 4°C, and supernatants were recovered and pre-cleared for non-specific binding proteins by incubation with blocked beads. To do this, control blocked beads (100 µL/5 mg protein) were washed three times in LB, added to the lysate and incubated at 4°C for 30 min with rotation. The bead slurry containing lysates was centrifuged at 10,000×g, the pre-cleared supernatant was removed and NaCl concentration adjusted to 1 M in preparation for affinity association.

Just before use, compound-immobilized beads (100 µL/5 mg protein) were washed 3 times with 1 mL pull-down buffer consisting of LB +1M NaCl (PDB) and resuspended in 2 bead volumes of the same buffer. Extract supernatant protein (3–5 mg analytical or up to 100 mg preparative) was added to the beads and incubated with rotation at 4°C for 45 min. The beads were washed 3 times with 10 bead volumes of PDB by incubating with rotation at 4°C for 5 min before each 10,000×g centrifugation and removal of the supernatant to eliminate unbound proteins. The wash procedure was repeated 3 additional times with LB. Elution was done with the addition of 2 bead volumes of LB supplemented with 0.56 mM SCYX-5070, 10 mM ATP and 20 mM MgCl_2_ with rotation at 4°C for 15 min. Proteins remaining bound to the beads were eluted by boiling in 2X Laemmli sample loading buffer. Aliquots of the total lysates and the various eluted samples were resolved on 4–12% Bis-Tris Criterion SDS-PAGE gels (Bio-Rad, Hercules, CA) and protein detection was done by either silver staining using SilverXpress staining kit (Invitrogen, Carlsbad, CA) or coomassie R-250 staining (Bio-Rad, Hercules, CA) for preparative separations that were followed by in-gel digestions for mass spectrometry analysis.

### Sample preparation for mass spectrometry

Individual gel bands were manually excised and subjected to overnight automated digestion with sequencing grade modified trypsin (Promega, Madison, WI) on a ProGest Digestor (Genomic Solutions, Ann Arbor, MI) at 37°C. Resultant peptides were lyophilized and re-dissolved in 5 µL of 50% methanol/0.1% trifluoroacetic acid (TFA). Peptides were spotted onto a MALDI target plate with an equal volume of α-cyano-4-hydroxycinnamic acid matrix solution and allowed to air dry.

### Mass spectrometry

Mass spectrometry was carried out on a 4800 Plus MALDI TOF/TOF Analyzer (Applied Biosystems, Foster City, CA). Peptides were scanned in positive reflector mode over the mass range 700–4000 m/z, with internal calibration against trypsin peaks 842.51 and 2211.105 m/z. The 40 most intense peptides were automatically selected for MS/MS analysis.

### Protein identification by database search

Peptide mass and corresponding MS/MS fragmentation information for each sample were searched against a database of *T. brucei* (http://www.genedb.org/genedb/tryp/index.jsp) or *L. major* (http://www.genedb.org/genedb/leish/) proteins using Mascot (Marix Science, Boston, MA) and GPS Explorer (Applied Biosystems, Foster City, CA). Proteins scoring above the Mascot significance threshold were considered as positive identifications.

## Results

### Parasite inhibition *in vitro*

**Figure 1:**
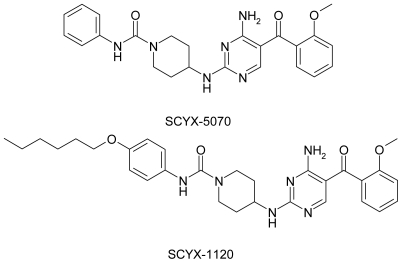
Chemical structures of SCYX-5070 and SCYX-1120.

SCYX-5070 and the related analogue SCYX-1120 (Figure 1) showed a dose-dependent killing of *T. brucei in vitro* as determined from the reduction of the resazurin marker for cell viability (Table 1). Blood stage and insect stage (procyclic) trypanosomes showed equal sensitivity towards both compounds. SCYX-5070 exhibited 63-fold selective inhibition of parasite versus mammalian cell (L929 mouse cell line) proliferation *in vitro* (Table 1). The corresponding selectivity for the less potent analogue SCYX-1120 was 38-fold. *Leishmania spp*. were less susceptible to killing by 2,4-diaminopyrimines, and the corresponding parasite versus host selectivity was also lower compared to that observed with *T. brucei* (Table 1). Dose-response curves generated from 2,4-diaminopyrimidine killing of *T. brucei* were relatively steep. For example, MIC values, determined as concentration at which no visible growth was observed after 72 h of exposure were 0.18 µM and 0.57 µM for SCYX-5070 and SCYX-1120, respectively.

**Table 1:** Activity of 2,4-diaminopyrimidine compounds against trypanosomatid protozoan parasites *in vitro.*

Cell/Parasite	Parasite Stage	SCYX-5070	SCYX-1120
*T. brucei*	Blood Stage	0.07±0.018	0.28±0.02
*T. brucei*	Procyclic	0.07±0.007	0.58±0.09
*L. donovani*	Promastigote	1.90±0.22	7.63±1.43
*L. major*	Promastigote	0.99±0.38	5.51±3.33
*L. donovani*	Amastigote	1.10±0.31	>10.81
L929 Fibroblast	Mammalian Cell	7.06±0.56	11.09±0.63

Compound sensitivity for each cell type was determined using resazurin as an indicator of viability following procedures described. Data are expressed as IC_50_ in µM and are averages from triplicate determinations.

### Time kill and tests for reversibility

**Figure 2:**
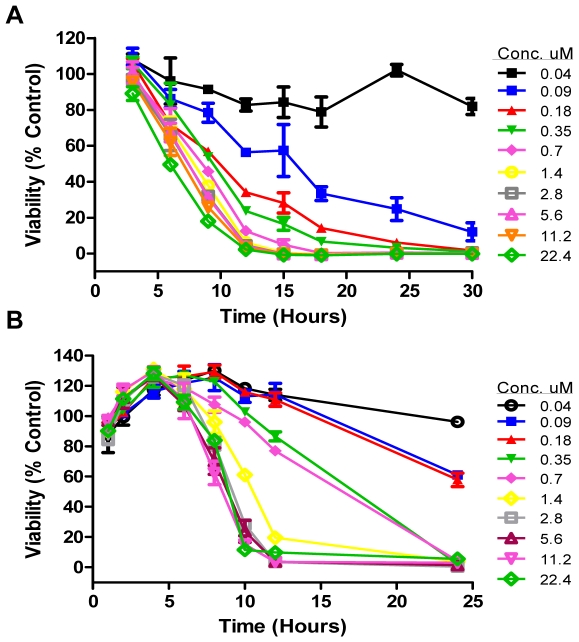
Time-to-kill plots demonstrating concentration-dependent and irreversibility of SCYX-5070 effects on *T. b. brucei in vitro*. Parasite survival was measured at various times in the presence on continuous drug (A) or after compound wash-out at specified times and viability assessment at 72 h (B). Experiments were conducted in triplicate and plots are mean survival of treated parasites expressed as percentages of control untreated parasites.

Studies of time-dose response (time kill) of *T. brucei* following exposure to SCYX-5070 are illustrated in Figure 2A. When measured 24 h after exposure, the IC_50_ value for SCYX-5070 killing of *T. brucei* is very close to that measured in the standard 72 h assay (0.09 vs 0.07 µM). Time-dependent loss of viability of *T. brucei* following exposure to SCYX-5070 was largely dependent upon compound concentrations up to 0.69 µM or approximately 4 times the MIC. Concentrations of SCYX-5070 above 0.69 µM did not result in faster or stronger killing of *T. brucei in vitro* (Figure 2A). A similar concentration-dependent and MIC-associated time-to-kill profile of *T. brucei* was observed for SCYX-1120, in which killing was maximal at 2.29 µM or 4 times the MIC (data not shown). Pulse incubation of *T. brucei* with SCYX-5070 shows that 10–12 h of compound exposure at concentrations above MIC is required to produce irreversible effects on parasite survival (Figure 2B). Persistency or irreversibility of SCYX-5070 effect is time and to a lesser degree, concentration dependent. As observed for time kill experiments, increasing concentration of compound far in excess of the measured MIC did not lead to more robust or faster killing after wash-out and incubation in compound-free medium. SCYX-1120 displayed reversibility kinetics that were similar to those observed with SCYX-5070-treated *T. brucei* parasites (data not shown).

### SCYX-5070 cures acute trypanosomiasis in the mouse model

The ability of SCYX-5070 to cure HAT in a mouse model was assessed using female Swiss Webster mice inoculated i.p. with 500 *T. brucei* parasites. Untreated control animals succumbed to the disease within 7–8 days following infection. SCYX-5070 administered i.p. twice daily at 20 mg/kg for 4 days starting 24 hours post-infection produced 100% cure rates with no evidence of compound related toxicity (Table 2). There was no dose related toxicity observed even when SCYX-5070 was dosed to infected animals at 50 mg/kg twice daily for 4 consecutive days (data not shown). Given that the ultimate goal of the program is to identify an orally active compound for the treatment of HAT, we also assessed the potential efficacy of SCYX-5070 in the mouse model following oral (p.o.) administration. SCYX-5070 cured all *T. brucei* infected animals when given po twice daily for 4 consecutive days (Table 2), suggesting good compound bioavailability.

**Table 2:** Treatment of *T. b. brucei* acute mouse infections^a^ with SCYX-5070.

Tested Compound	Dose^b^ (mg/kg)	Dosing Route	Cured^c^/Total	Ar. Survival (Days)	% Cured^c^
SCYX-5070	20	IP	3/3	>30	100
SCYX-5070	20	PO	3/3	>30	100
Pentamidine	2	IP	3/3	>30	100
Untreated	0	-	0/3	7	0

a Mice were infected with 500 *T. b. brucei* EATRO 110 parasites and the infection was allowed to progress for 24 hrs prior to initiation of treatment. Parasitemia was examined weekly and cures were determined as described in materials and methods.

b Infected animals were dosed twice daily for 4 consecutive days. Pentamidine was administered once daily for 4 days to serve as positive control.

c Cured animals are defined as those that remained parasite free >30 days after the start of the dosing period.

### Affinity purification and identification of protein targets

**Figure 3:**
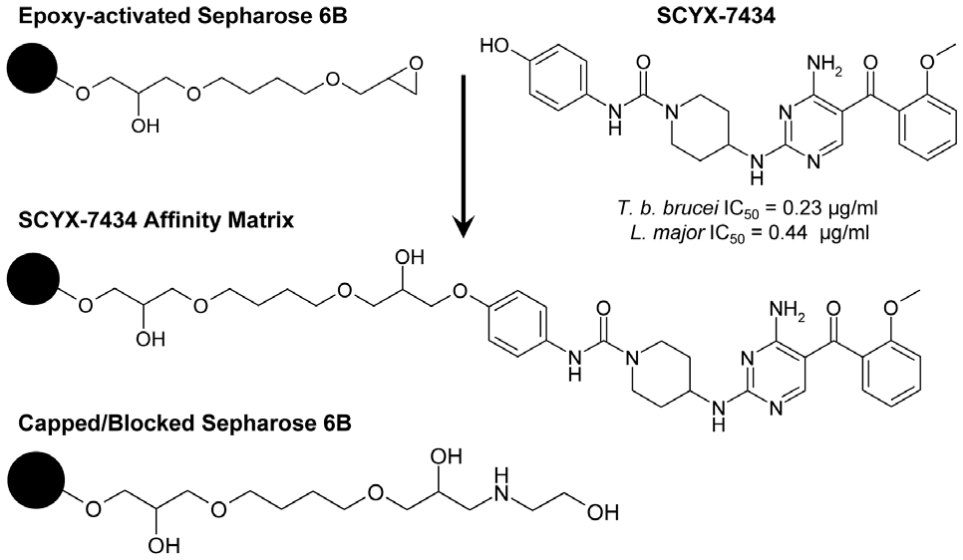
Chemical structure of free and immobilized 4-[4-Amino-5-(2-methoxy-benzoyl)-pyrimidin-2-ylamino]-piperidine-1-carboxylic acid (4-hydroxy-phenyl)-amide (SCYX-7434). SCYX-7434 was coupled to activated sepharose 6B via the carbodiimide reaction, as described in materials and methods. Control capped sepharose 6B was produced by the addition of ethanolamine to block the reactive groups.

**Figure 4:**
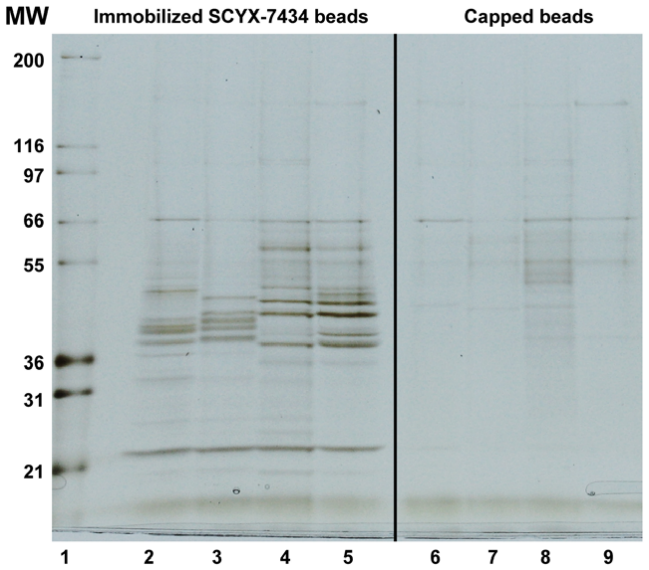
Trypanosomatid protozoan proteins interacting with immobilized diaminopyridine. Soluble extracts from different stages of *T. brucei* and *Leishmania spp.* were loaded onto compound-immobilized or control sepharose beads. Proteins bound to each matrix were eluted and resolved on SDS-PAGE followed by visualization with silver staining. Protein samples are: molecular weight markers (lane 1); *L. donovani* promastigotes (lane 2, 6); *L. major* promastigotes (lane 3, 7); *T. brucei* procyclics (lane 4, 8) and *T. brucei* blood stage (lane 5, 9).

To generate an appropriate ligand for affinity immobilization, an analogue of SCYX-5070 containing a hydroxyl, SCYX-7434, was synthesized and coupled to ECH Sepharose (Figure 3). To determine that the position used for immobilization does not result in loss of activity, compound SCYX-1120 (Table 1) was synthesized with an extended carbon linker off the phenyl ring. SCYX-1120 retained significant *in vitro* activity against *T. b. brucei* (Table 1), indicating that it retained SCYX-5070-like binding properties. To identify potential protein targets that interact with SCYX-5070 and related compounds, total extracts prepared from *T. brucei* or *Leishmania spp.* were incubated with immobilized SCYX-7434 or control capped sepharose beads. Following extensive washing, the bound proteins were eluted with free SCYX-5070 and ATP. Visualization of the eluted samples by SDS-PAGE and silver staining showed several proteins, most being in the 36–50 kD range (Figure 4, lanes 2–5). Most of the proteins interact specifically with SCYX-7434-containing beads, as indicated by the absence of significant staining in eluates from capped beads (Figure 4, lanes 6–9). The affinity matrix interacts with proteins of similar molecular sizes in procyclic (Figure 4, lane 4) and blood stream form *T. brucei* (Figure 4 lane 5), although there are differences in the intensities of some bands. Similarly, the major protein bands released with free compound and ATP from affinity matrices loaded with extracts from promastigotes of *L. major* (Figure 4, lane 3) and *L. donovani* (Figure 4, lane 2) were similar with some minor exceptions. Collectively, the eluted protein profile from association experiments suggests that the immobilized 2,4-diaminipyrimidine is recognizing similar cellular targets in trypanosomatid protozoans examined in these studies.

**Figure 5:**
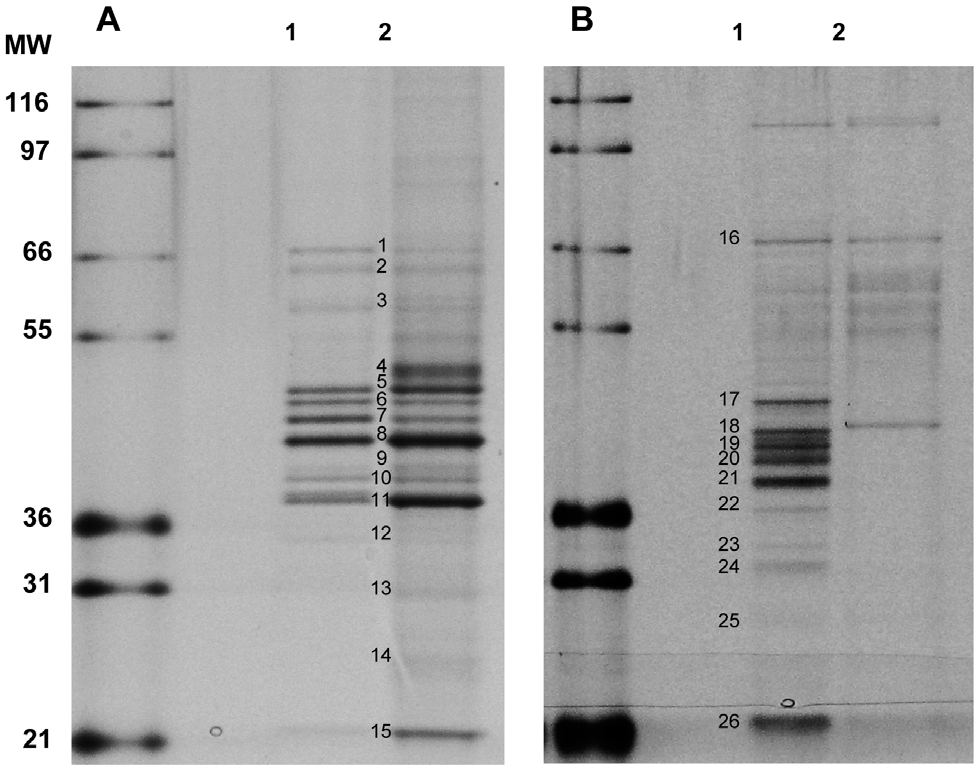
Analysis and identification of proteins retained by immobilized SCYX-7434. Lysates from blood stage *T. brucei* or *L. major* were loaded onto SCYX-7434-immobilized sepharose beads, eluted and analyzed on silver stained SDS-PAGE gels. (A) Bound *T. brucei* proteins were eluted with SCYX-5070 and ATP (lane 1) or by boiling in sample buffer (lane 2). (B) *L. major* proteins bound to immobilized SCYX-7434 (lane 1) or capped control beads (lane 2) were eluted with SCYX-5070 and ATP.

To identify potential protein targets for 2,4-diaminopyrimidines, preparative amounts (up to 100 mg) of lysates from *T. brucei* or *L. major* were incubated with immobilized SCYX-7434, eluted with free compound and ATP followed by SDS-PAGE analysis. In some instances, proteins that remained on the beads after the initial elution were dissociated by boiling the beads in SDS-PAGE loading buffer to provide sufficient material for downstream protein analysis and identification work. Eluted proteins were separated on SDS-PAGE gels and visualized by coomassie blue staining. Protein bands were excised from the stained gel, subjected to tryptic digestion and analysis by MALDI-TOF/TOF spectrometry. A small aliquot from each of the preparative elutions was retained for analysis on SDS-PAGE and silver staining to provide a higher resolution image (Figure 5A and B) to correlate with and aid in the interpretation of coomassie stained bands that were subjected to MALDI-TOF/TOF analysis. A comprehensive listing of all proteins that were positively identified in this analysis is shown in Table S1. Table 3 shows the key target proteins that are common to both *T. brucei* and *L. major* and they include MAPKs, CRKs and other non-kinase proteins (Figure 5; Table 3). MAPK2 and 9 were isolated from both *T. brucei* (Figure 5A) and *L. major* (Figure 5B). In contrast, CRKs 1 and 6 were identified in *T. brucei* (Figure 5A) while CRK3 was identified in *L. major* (Figure 5B). Surprisingly, each of the MAPK proteins identified migrated as multiple bands on SDS-PAGE gels. This was observed in samples obtained from both *T. brucei* and *L. major* association experiments. Proteins eluted from samples prepared from *L. donovani* were not subjected to MALDI-TOF/TOF analysis as there is currently no genome database for this species of *Leishmania spp*.

**Table 3:** Protein targets identified from *T. brucei* and *L. major* by LC/MS/MS.

aProtein #	Name	bAccession	cMW (Da)
1	HSP70 (Heat-shock protein 70), MAPK14	Tb11.01.3110, Tb927.3.690	75320, 63787
2	MAPK14	Tb927.3.690	63787
3	MAPK14	Tb927.3.690	63787
4	Tubulin, α and β	Tb927.1.2360, Tb927.1.2350	49755, 49672
5	MAPK2, EF-1α (Elongation factor 1-alpha)	Tb10.70.2070, Tb10.70.5680	49710, 37784
6	MAPK2, MAPK14	Tb10.70.2070, Tb927.3.690	49710, 63787
7	MAPK2, MAPK9	Tb10.70.2070, Tb10.61.1850	49710, 42723
8	MAPK2, MAPK9	Tb10.70.2070, Tb10.61.1850	49710, 42723
9	MAPK2, MAPK9	Tb10.70.2070, Tb10.61.1850	49710, 42723
10	MAPK2, MAPK9, CRK6	Tb10.70.2070, Tb10.61.1850, Tb11.47.0031	49710, 42723, 36705
11	MAPK9, CRK6	Tb10.61.1850, Tb11.47.0031	42723, 36705
12	MAPkinase kinase kinase, CRK1	Tb01.61.1880, Tb10.70.7040	31355, 34329
13	MAPK9	Tb10.61.1850	42723
14	MAPK9, Tryparedoxin peroxidase	Tb10.61.1850, Tb09.160.4280	42723, 22410
15	Cyclophilin A	Tb11.03.0250	18705
16	HSP70	LmjF28.2780	71608
17	EF-1α	LmjF17.0083, LmjF03.0820	49085
18	MPK2	LmjF36.0720	48309
19	MPK2	LmjF36.0720	48309
20	MPK2	LmjF36.0720	48309
21	MPK9, putative protein kinase	LmjF19.0180, LmjF34.0030	44964, 44583
22	CRK3	LmjF36.0550	35622
23	Antigen	gi|6531419	30016
24	40S Ribosomal protein S4	LmjF13.1230	30663
25	Tryparedoxin peroxidase	LmjF15.1080	22114
26	Cyclophilin A	LmjF25.0910	18811

a Protein number matches annotations in figure 5A and B.

b Accession numbers and

c molecular weight obtained from genome databases.

**Figure 6:**
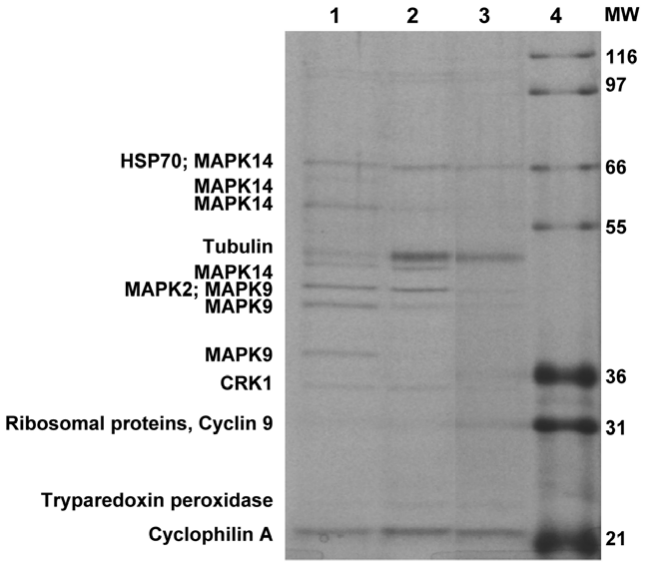
Competitive lysate binding in the presence of free SCYX-5070. Procyclic *T. brucei* lysates were bound in the presence of varying concentrations of SCYX-5070. No SCYX-5070 (lane 1), 2.5 µM SCYX-5070 (lane 2), 40 µM SCYX-5070 (lane 3).

To further probe the specificity of the kinase and non-kinase proteins identified, SCYX-7434 association experiments with *T. brucei* extracts were conducted in the presence or absence of varying concentrations of free SCYX-5070. The presence of 2.5 µM SCYX-5070 resulted in the loss of several bands representing MAPK9 and 14 (Figure 6). When the concentration of SCYX-5070 was increased to 40 µM in the association experiments, MAPK 2 and CRK1 were no longer retained on the beads (Figure 6). The association of immobilized SCYX-7434 with non-kinase proteins HSP70, tubulin, ribosomal proteins, tryparedoxin peroxidase and cyclophilin A were not affected by the presence of SCYX-5070 up to 40 µM. (Figure 6).

## Discussion

Current HAT treatment regimens consist of pentamidine, suramin, melarsoprol or eflornithine. The potential for effective treatment of the disease using these drugs is very limited because they are toxic or require cumbersome parenteral administration [2]–[5]. With over 50 million people at risk of HAT in sub-Saharan Africa, the need for a safe, effective and easily administered treatment is crucial. Here we report that SCYX-5070 is a potent inhibitor of *T. brucei* growth and exhibits good physicochemical properties to serve as a lead for development of new drug candidates for treatment of HAT. SCYX-5070 is a 2,4-diaminopyrimidine related to a class of potent ATP competitive inhibitors of mammalian CDKs, which have been explored as potential treatments for cancer [7]. In this work, we demonstrate that MAPKs and CRKs are the likely targets mediating the mechanism of trypanocidal activity of SCYX-5070 and related 2,4-diaminopyrimidines. While several *T. brucei* kinases have been indirectly implicated as essential enzymes, and thus potential drug targets, we are not aware of examples of any small molecule kinase inhibitors for the treatment of HAT or any other disease of protozoan origin.

*In vitro* sensitivity assays showed potent trypanocidal activity of SCYX-5070 and other related 2,4-diaminopyrimidines with parasite versus host cell selectivity >60-fold. This level of selectivity suggests a low potential for toxicity towards host cells and provides an early indication of differences between parasite versus mammalian target proteins. Closely related 2,4-diaminopyrimidines are known to target mammalian CDKs [7], [8] while another class of 2,4-diaminopyrimidines related to trimethoprim was shown to target DHFR [21]–[23]. Interestingly, the different life-cycle stages of both *T. brucei* and *L. donovani* displayed the same level of sensitivity to 2,4-diaminopyrimidines reported in our current work. This observation suggests that the molecular targets through which SCYX-5070 exerts anti-parasitic effects are not developmentally limited. Phenotypic analyses of treated parasites did not reveal a specific kinase or cell-cycle related effect. The presence of potential targets in all stages of the parasites enabled us to use the insect stages of *Leishmania spp.* (promastigotes) and *T. brucei* (procyclics) for protein isolation since these stages are relatively easy to culture and can provide large quantities of protein required in the optimization of association experiments with SCYX-7434.

Time kill studies with SCYX-5070 yielded a concentration- and time-dependent decrease in ATP content or viability of *T. brucei* blood stage parasites. Maximal trypanosome kill rates were observed within 12–15 h following exposure of SCYX-5070 at concentrations 4 times the MIC. These data suggest that 2,4-diaminipyrimidines are primarily time-dependent trypanocidal agents, the efficacy of which is only partly dependent on concentration. Because the compounds are highly permeable, the apparently saturable effects at high concentration suggest that the target is limiting. In addition, P2 transporter knockout and wild-type showed similar levels of sensitivity towards SCYX-5070. Studies of the reversibility of trypanocidal effects revealed that exposure of *T. brucei* to SCYX-5070 at concentrations 8–10-fold above the MIC for 10–12 h results in elimination of parasites from culture even after the compound has been washed out. The inability of *T. brucei* parasites to recover from transient exposure to compound *in vitro* suggests that 2,4-diaminopyrimidines are retained within the parasites or that they exert irreversible effects on the potential target(s) within the parasite during this time. This rapid and irreversible kill rate and microscopic examinations suggest that 2,4-diaminopyrimidines are cidal agents against *T. brucei.* Encouraged by the potency and time-kill profile of SCYX-5070 and related 2,4-diaminopyrimidines *in vitro*, studies were conducted to determine the potential of this class of compounds to cure animals infected with *T. brucei*. Cure (100%) of an acute *T. brucei* infection in a mouse model was accomplished with a 4 day regimen to complement the potency observed from *in vitro* experiments. Efficacy *in vivo* against *T. brucei* suggests that SCYX-5070 has sufficient stability and pharmacokinetics to warrant further exploitation as a potential treatment for HAT. Furthermore, SCYX-5070 did not exhibit toxic side effects when it was administered to *T. brucei* infected mice at levels (50 mg/kg) far in excess of what was required for full efficacy (20 mg/kg) in a 4-day treatment regimen.

Although compounds from the 2,4-diaminopyrimidine class related to SCYX-5070 are potent and selective inhibitors of mammalian CDKs, we show that analogues selective for parasite killing versus mammalian host can be developed. These observations suggest the potential for different or novel targets in parasitic protozoa. Using a chemical proteomics approach involving immobilized compound affinity chromatography followed by MALDI-TOF/TOF and database searching, we identified *T. brucei* MAPKs and/or CRKs as the likely targets mediating anti-parasitic activity of this class of kinase inhibitors in protozoan parasites. This is in contrast to previous studies that demonstrated that 2,4-diaminopyrirmidines closely related to SCYX-5070 do not inhibit mammalian (human) MAPKs even when tested up to 50 µM [8].

MAPKs from *T. brucei* and *L. major* were recovered as multiple bands when association and pull-down experiments were conducted with immobilized SCYX-7434 (Figure 5A and B). The multiple banding phenomenon was not observed with CRKs or other non-kinase proteins that were recovered from the affinity matrix. This finding suggests that the multiple bands of MAPKs are not the result of general proteolysis that may occur during parasite lysis and sample preparation. Analysis of all the identified peptide fragments for the various affinity-purified MAPKs revealed that fragments are the result of varying degrees of C-terminus truncation that match the approximate band size observed on the SDS-PAGE gels. A similar multiple banding of MAPKs has been observed previously when *Leishmania spp.* proteins were analyzed by tryptic digestion and mass spectrometry (G. Spath, personal communication). In a recent study, a proteomic analysis of procyclic *T. brucei* also noted that several proteins were present in multiple variant forms [24]. This phenomenon has been suggested as a potential mechanism that enables the parasites to control activity of the protein in question. The precise mechanism that leads to the generation of multiple forms of MAPKs is unknown, but it seems to be a relatively common observation on trypanosomatid proteins.

Various non-kinase proteins from *T. brucei* or *L. major* such as tubulin, fructose aldolase, ribosomal proteins, cyclophilin, HSP70, GAPDH and EF1-α were also observed to interact with the SCYX-7434 affinity matrix. However, we and others suggest that binding of these proteins is not specific to the 2,4-diaminopyrimidine or other kinase affinity ligands [25]–[27]. Some of these proteins may also be associated with the matrix simply because they are abundant within the extracts prepared from protozoan parasites or other sources. For example, tubulin is a highly abundant protein that also associates with SCYX-7434 affinity matrix as well as the capped control beads, and this has been observed in similar affinity work by others [25]–[27]. Other highly expressed proteins that readily associate with affinity matrices used in kinase isolations include HSP70, EF 1-α, GAPDH, ribosomal proteins and fructose aldolase [16], [24], [25]. In our work, most of the non-kinase proteins including cyclophilin and tryparedoxin peroxidase remained bound to the SCYX-7434 affinity beads when free SCYX-5070 was included in the association buffer. This was in contrast to MAPKs and CRKs, which could be selectively inhibited from the affinity matrix by excess free SCYX-5070, suggesting a more specific interaction with immobilized SCYX-7434.

To ensure that kinases were not associating with the SCYX-7434 simply because of their relative abundance, we compared gene expression levels for the MAPK and CRK family of proteins. This was done by mining published data sets from *T. brucei* genome wide expression studies [28], [29]. From this analysis, we determined that MAPK 2, 9 and 14 are in the lower to middle half of expression levels of the MAPK family. Interestingly, MAPK 2 is one of the lowest expressed members but it was one of the most prominent proteins identified from *T. brucei* extracts in our work. Examination of the CRK family in the genome expression database indicates that CRK6 is highly expressed (compared to other CRKs) in the bloodstream form of *T. brucei* and levels drop about 3 fold in procyclics. Expression data for CKR1 was divergent between the two studies relative to the other family members. The significance of these expression levels is unclear since the relation between mRNA and protein levels for these specific kinases are unknown at present. Nevertheless, the information on relative expression levels versus recovery from association experiments could be valuable in prioritization of targets for subsequent validation efforts.

The MAPKs identified in this work as potential targets for SCYX-5070 are highly conserved eukaryotic proteins involved in cellular processes such as proliferation, motility, cell-shape, stress response, apoptosis and differentiation [30]. The trypanosomatid protozoa *Leishmania* and *Trypanosoma* go through complex biochemical and morphological changes during their life cycle, which alternates between an insect vector and a mammalian host. The *T. brucei* genome contains at least 13 MAPKs (MAPK 1–6; 9–15) while *Leishmania spp.* has at least 15 (MAPK 1–15) [30]. In our work, *T. brucei* MAPK 2, 9 and 14 were identified as targets of association with SCYX-5070. Functional studies are not available for any of the *T. brucei* MAPKs targeted by SCYX-5070 or related 2,4-diaminopyrimidines. In contrast, both MAPK targets of SCYX-5070 in *Leishmania*, MAPK 2 and 9, have been shown to be essential or play a role in the biology of the parasite [30]. For example, MAPK 2 from *L. mexicana* is required to establish an infection in the mouse model for leishmaniasis [30]. MAPK 9 deletion mutants have significantly elongated flagella and over-expression has the opposite effect [31].

*T. brucei* has 11 CRKs (CRK1-4 and CRK6-12) most of which seem to have evolved substantial parasite-specific features [32]. In addition, 10 cyclins (CYC2-11) have been identified in *T. brucei*, though very little is known about their pairings to CRKs except for CRK 3, which interacts with CYC2 and CYC6 [33], [34]. Trypanosomatid CRKs are phylogenetically related to CDKs from various organisms, but most of them have insertions or other modifications in their primary structure, suggestive of potential novel mechanisms of regulation of activity [32]. Two of the three CRKs (i.e. CRK 1 and CRK 3), identified as potential targets for SCYX-5070 in this work, have been implicated as key regulators of G1/S and G2/M checkpoint [35]. These observations involving RNAi knockdowns show a complete block of cell growth or total G2/M arrest only when knockdown of CRK 3 was paired with that of another CRK (i.e. CRK 1, 2, 4 or 6). It is conceivable that inhibition of multiple CRKs by SCYX-5070 is required for maximal suppression of *T. brucei* growth or survival *in vivo*. Chemical validation of CRK3 in *Leishmania spp.* was demonstrated by screening of a chemical library against a recombinant *L. donovani* CRK 3. The work identified 2,6,9-trisubstituted purines, including the C-2-alkynylated purines, the indirubins, the paullones and derivatives of the non-specific kinase inhibitor staurosporine as some of the key hits [36]. Unfortunately, these compounds lacked selectivity for parasite versus mammalian CDKs and could not be exploited any further.

In summary, we have showed the activity of 2,4-diaminopyrimidines (SCYX-5070) against *T. brucei* growth *in vitro*. These compounds also display robust activity in animal models of acute HAT. We have used a proteomics approach to demonstrate MAPKs and CRKs as potential targets mediating the mechanism of action of SCYX-5070 and related compounds against trypanosomatid protozoa. The effectiveness of 2,4-diaminopyrimidines as anti-trypanosome agents *in vitro* and *in vivo* provide a potential good starting point for the development of a much needed safe and effective new treatment for sleeping sickness.

## Supporting Information

Table S1 Protein identification by database search.
(0.03 MB XLS)
